# Hydrophilic oxygen radical absorbance capacity values of low-molecular-weight phenolic compounds containing carbon, hydrogen, and oxygen

**DOI:** 10.1039/d1ra08918h

**Published:** 2022-02-02

**Authors:** Shuhei Sakurai, Akito Kikuchi, Hiroaki Gotoh

**Affiliations:** Department of Chemistry and Life Science, Yokohama National University 79-5 Tokiwadai, Hodogaya-ku Yokohama 240-8501 Japan gotoh-hiroaki-yw@ynu.ac.jp +81-45-339-3964 +81-45-339-3964

## Abstract

The antioxidant capacity of an antioxidant reflects its ability to remove reactive oxygen species (ROS). In this study, the hydrophilic oxygen radical absorbance capacity (H-ORAC) method was used to quantitatively evaluate the antioxidant capacities of natural phenols and their derivatives against peroxyl radicals. This method was comprehensively applied to low-molecular-weight phenols to construct a database. Although no macroscopic correlation was observed for values related to the antioxidant capacity expression, we observed a difference in the trend of the H-ORAC values for each functional group. Thus, this database will serve as a new benchmark and tool for molecular design.

## Abstract 2

The hydrophilic oxygen radical absorbance capacity (H-OARC) assay measures the antioxidant capacity of compounds against the peroxyl radical, a reactive oxygen species.

## Graphical abstract

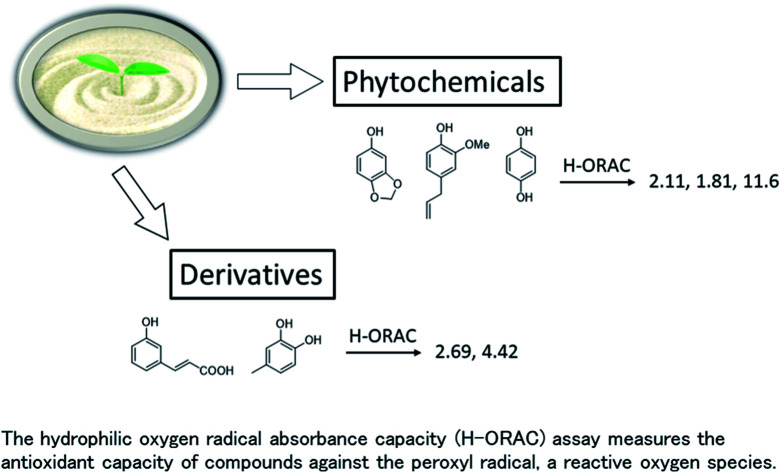

## 1. Introduction

In organisms, the oxygen consumed by breathing is converted to reactive oxygen species (ROS), including hydroxyl radicals, peroxides, singlet oxygen species, peroxy radicals, and hydroperoxide, and this process is promoted by external stimuli.^1^ However, these ROS can be neutralized by antioxidants, wherein the antioxidant capacity is defined as the ability of an antioxidant to either inhibit the generation and function of ROS or remove them.

The oxygen radical absorbance capacity (ORAC) assay is a key method employed to measure the antioxidant capacities of compounds against peroxyl radicals. In this method, the antioxidant capacity is measured by evaluating the inhibition of the fluorescent probe degradation by the peroxyl radicals produced from 2,2′-azobis(2-amidinopropane) dihydrochloride (AAPH).^2^ In this method, radical species that mimic lipid peroxyl radicals are generated, and the fluorescence decay over time is measured. Additionally, this reaction is performed in a phosphate buffer, which possesses a pH close to that of the human body, and so this system can essentially replicate the oxidation reaction taking place in the body.^3^

The ORAC method can be applied to measure the antioxidant capacities of biological samples, such as serum,^4^ although the antioxidant capacities of hydrophilic (H-ORAC)^5,6^ and lipophilic (L-ORAC)^6,7^ antioxidants can also be measured, depending on the solution added to the phosphate buffer. ORAC is a typical method for measuring the antioxidant capacity and is widely used in the evaluation of a variety of foods and food components.^8–10^

Depending on the type of antioxidant, the decay behavior of the fluorescence intensity can change in the ORAC measurement. In this context, a previous study reported an induction or lag time before the decrease in the fluorescence intensity, with some compounds exhibiting a rapid decrease after the lag time (*e.g.*, trolox or *trans*-ferulic acid) or a gradual decrease without the lag time (*e.g.*, catechin).^11^ This phenomenon is observed due to the structural differences between the antioxidants, which allow them to react rapidly or persistently with peroxyl radicals. Although these structural differences have been attributed to bond dissociation energies and steric influences,^12^ the specific details remain unknown.

Considering the above points, comprehensive data are therefore required to analyze the correlation between the molecular structure and the activity using statistical methods. However, the amount of data currently reported is insufficient, and to the best of our knowledge, there is no example of measuring more than 30 components within the same study. Additionally, the accuracy varies depending on the reporter. More specifically, it has been indicated that the original H-ORAC method exhibited problems in terms of reproducing the obtained values,^5^ with some cases reporting different ORAC values for the same compound. For example, values of both 5.3 and 11.34 mol TE mol^−1^ were reported for the quercetin molecule.^13,14^ Such variations therefore render it challenging to compare the reported cases.

In this study, we report a comparable database of ORAC measurements for natural phenols and their derivatives using the H-ORAC method.^5^ The reproducibility of this method is confirmed through interlaboratory collaborative studies, and the structural characteristics of various antioxidants were clarified based on these data.

It should be noted here that the antioxidant capacity can be determined from the structural features of a compound using computational chemistry to estimate the physical properties and reactivity in terms of the relevant mechanisms. Generally, phenolic compounds exhibit an antioxidant activity against the peroxyl radical *via* the hydrogen transfer mechanism (hydrogen atom transfer; HAT (eqn (1)),^15^ the electron transfer mechanism (electron transfer-proton transfer; ET-PT (eqn (2.1) and (2.2)),^16^ and sequential proton loss electron transfer (SPLET (eqn (3.1) and (3.2)).^17^

1 ArOH + ROȮ → ArȮ + ROO–H

2.1 ArOH + ROȮ → ArOḢ^+^ + ROO^−^

2.2 ArOḢ^+^ + ROO^−^ → ArȮ + ROO–H

3.1 ArOH → ArO^−^ + H^+^

3.2 ArO^−^ + ROȮ → ArȮ + ROO^−^

Although the expression of the antioxidant capacity in the ORAC measurements is considered to proceed *via* the HAT^1^ or ET-PT mechanism,^3,18^ it may also proceed *via* the SPLET mechanism.^4,19^ Hence, we herein calculate the physical properties of the phenolic compounds using quantum chemical calculations to determine the dominant mechanism. In addition, we estimate the effect of each functional group by examining changes in the ORAC value.

## 2. Experimental

### 2.1 Reagents

The reagents selected for use in this study were composed of only of carbon, hydrogen, and oxygen. All reagents were purchased from Tokyo Chemical Industry, FUJIFILM Wako Chemicals, Junsei Chemical Co., Ltd, or Sigma-Aldrich. Phenolic compounds classified by the terms “phytochemical” and “botanical” were selected from the PubChem database, along with their corresponding analogs and derivatives. In particular, the selected compounds were phenolic compounds bearing methyl, ethyl, propyl, butyl, amyl, methoxy, hydroxy, aldehyde, *t*-butyl, and iso-propyl groups, which are readily available reagents. The partially ketonic carbon chains were also included. In addition, benzaldehyde and 2,4-dimethoxybenzaldehyde were also examined as other compounds.

### 2.2 H-ORAC measurements

The H-ORAC measurements were carried out according to a previously described method.^5^ AAPH, fluorescein, and trolox were used as the radical generator, labeling agent, and reference material, respectively. A standard solution was prepared for each target compound (1 mg mL^−1^) in methanol : water : acetic acid (90 : 9.5 : 0.5), and the solution pH was adjusted to 7.4 using a phosphate buffer solution. The measurements were performed following the standard operating procedures, and the results were converted to trolox equivalents (TE mol mol^−1^). Skanlt RE for Varioskan flash 2.4 was used as the plate reader. The H-ORAC value was quoted as the average of the converted values obtained from three measurements for each compound. The measurement was considered invalid when ferulic acid was simultaneously measured, and the corrected value was outwith a value of 17 552 ± 1864 μmol TE L^−1^ (3.408 ± 0.362 TEmol mol^−1^).

### 2.3 Computational chemistry

Dragan *et al.*^20^ reported that parameterization method 6 (PM6)^21^ has an accuracy similar to that of the density-functional theory approach^22^ in numerical calculations related to radical reactions. Hence, we used PM7 (ref. 23) to perform the calculations. The most stable structure for each compound was determined by exploring its coordination based on the molecular force field calculations using Balloon.^24^ The most stable structure was employed as the initial structure for subsequent calculations, and semi-empirical calculations were performed using the Molecular Orbital PACkage.^25^ The PM7 method was applied to optimize the structure under vacuum conditions. The enthalpies obtained from the calculations were employed to calculate the thermodynamic parameters using the following equations:BDE = *H*(ArȮ) + *H*(Ḣ) − *H*(ArOH)IP = *H*(ArOḢ^+^) + *H*(e^−^) − *H*(ArOH)PDE = *H*(ArȮ) + *H*(H^+^) − *H*(ArOḢ^+^)PA = *H*(ArO^−^) + *H*(H^+^) − *H*(ArOH)ETE = *H*(ArȮ) + *H*(e^−^) − *H*(ArO^−^)

Here, an *H*(e^−^) value of 0.7516 kcal mol^−1^ was used as the enthalpy in a vacuum.^26^ The bond dissociation enthalpy (BDE) was selected to support the HAT mechanism, the adiabatic ionization potential (IP) and the proton dissociation enthalpy (PDE) were selected to support the ET-PT mechanism, and the proton affinity (PA) and electron-transfer enthalpy (ETE) were selected to support the SPLET mechanism. For determination of the BDE and PA values, the hydrogen bond with the smallest value was considered to react most readily with the peroxyl radical.

## 3. Results and discussion

**Fig. 1:**
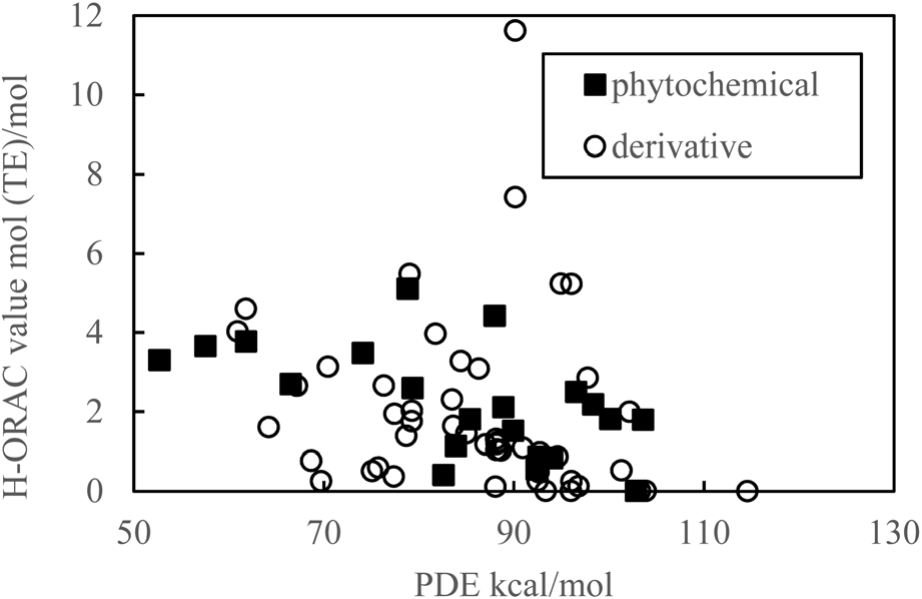
Scatter plot of the proton dissociation enthalpy (PDE) against the hydrophilic oxygen radical absorbance capacity (H-ORAC) value.

**Fig. 2:**
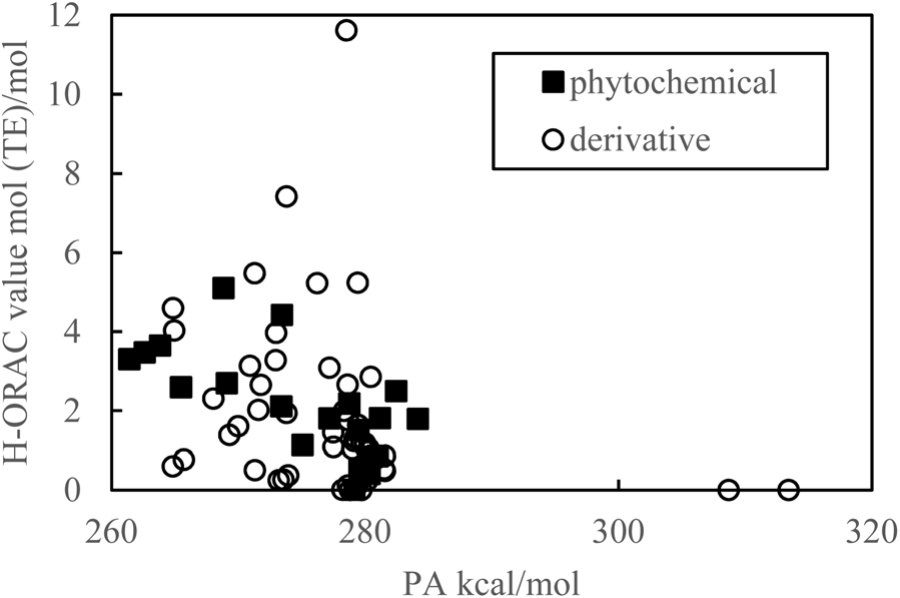
Scatter plot of the proton affinity (PA) against the hydrophilic oxygen radical absorbance capacity (H-ORAC) value.

**Fig. 3:**
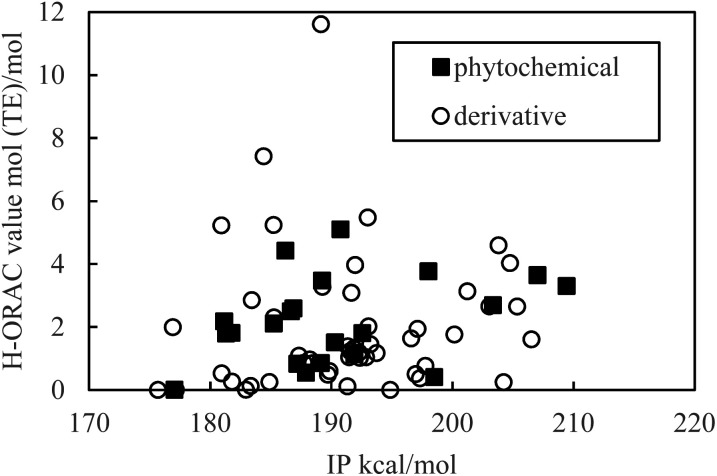
Scatter plot of the adiabatic ionization potential (IP) against the hydrophilic oxygen radical absorbance capacity (H-ORAC) value.

**Fig. 4:**
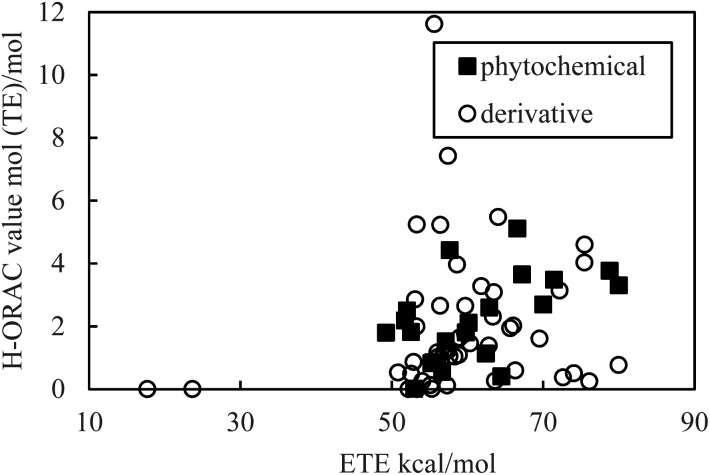
Scatter plot of the electron-transfer enthalpy (ETE) against the hydrophilic oxygen radical absorbance capacity (H-ORAC) value.

As indicated in Fig. 1 and 2, the PDE and PA values do not correlate with the H-ORAC values because the reaction occurs in a neutral environment with the involvement of protons. Similarly, the IP and ETE values do not correlate with the H-ORAC values because the reactions occur in a neutral environment with the involvement of electrons, while the steric factors of the molecules do not participate (Fig. 3 and 4).

**Fig. 5:**
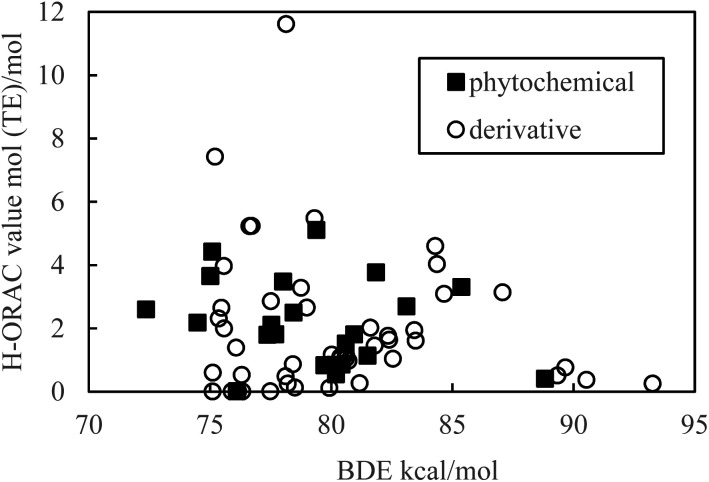
Scatter plot of the bond dissociation enthalpy (BDE) against the hydrophilic oxygen radical absorbance capacity (H-ORAC) value.

Hydrogen is easily withdrawn when the BDE is small, thereby increasing the reactivity with ROO. Therefore, it is inferred that there is a negative correlation between a small BDE and a H-ORAC value, even though there is no correlation in reality (Fig. 5).

Although the macroscopic correlation is inefficient, as described above, the trend in the H-ORAC values can be demonstrated by examining the H-ORAC values between molecules with similar structures. Thus, the H-ORAC values and the BDE, IP, PDE, PA, and ETE values are presented in five different scatter plots, as summarized in Table 1. It should be noted that no macroscopic correlations were observed between these values and the H-ORAC values.

**Table 1:** Select values related to the hydrophilic oxygen radical absorbance capacity (H-ORAC) value

IUPAC name (common name)	H-ORAC (mol TE mol^−1^)	Standard deviation (mol TE mol^−1^)	Bond dissociation enthalpy (kcal mol^−1^)	Ionization potential (kcal mol^−1^)	Proton dissociation enthalpy (kcal mol^−1^)	Proton affinity (kcal mol^−1^)	Electron-transfer enthalpy (kcal mol^−1^)
Benzene-1,4-diol (hydroquinone)	11.614	0.438	78.15	189.16	90.14	278.55	55.63
4-*tert*-Butylbenzene-1,2-diol	7.418	0.321	75.22	184.42	90.16	273.82	57.43
2,5-Dihydroxybenzoic acid	5.475	0.123	79.32	193.02	79.03	271.29	64.06
2-Methylbenzene-1,4-diol(methylhydroquinone)	5.235	1.122	76.73	185.25	94.95	279.44	53.32
2-Methoxybenzene-1,4-diol(methoxyhydroquinone)	5.226	1.608	76.63	180.93	96.07	276.24	56.42
1-(4-Hydroxyphenyl)butan-1-one(4′-hydroxybutyrophenone)	4.595	0.997	84.30	203.82	61.79	264.85	75.48
1-(4-Hydroxyphenyl)ethanone(4′-hydroxyacetophenone)	4.025	0.586	84.37	204.76	60.94	264.95	75.46
Benzene-1,2-diol	3.965	0.240	75.58	191.98	81.76	272.99	58.63
1-(2,5-Dihydroxyphenyl)ethanone (2′,5′-dihydroxyacetophenone)	3.274	0.235	78.76	189.27	84.43	272.95	61.84
Benzene-1,3,5-triol(phloroglucinol)	3.133	0.240	87.08	201.24	70.44	270.93	72.18
3-Methoxyphenol	3.084	0.369	84.66	191.66	86.30	277.21	63.49
4-Methoxyphenol	2.852	0.123	77.51	183.45	97.77	280.47	53.08
2-Hydroxybenzaldehyde	2.653	0.472	79.00	203.06	76.33	278.65	56.39
3-Hydroxybenzaldehyde	2.648	0.528	75.47	205.36	67.18	271.78	59.73
Benzene-1,2,4-triol	2.305	0.124	75.37	185.28	83.51	268.04	63.37
1-(4-Hydroxyphenyl)propan-2-one(4-hydroxyphenylacetone)	2.015	0.159	81.63	193.08	79.28	271.61	66.05
2-*tert*-Butyl-4-methoxyphenol	1.990	0.243	75.59	176.93	102.18	278.36	53.26
Benzene-1,3-diol	1.941	0.271	83.44	197.13	77.44	273.81	65.66
Phenol	1.758	0.160	82.35	200.17	79.26	278.68	59.71
3-Methylphenol	1.635	0.091	82.40	196.60	83.62	279.47	58.97
3-Hydroxybenzoic acid	1.604	0.256	83.49	206.53	64.22	270.00	69.53
2-Prop-2-enylphenol(2-allylphenol)	1.453	0.061	81.80	193.24	85.01	277.50	60.33
Benzene-1,2,3-triol	1.384	0.258	76.08	191.38	78.67	269.30	62.81
4-Propylphenol	1.312	0.231	80.60	191.87	88.14	279.26	57.37
4-Butylphenol	1.232	0.177	80.60	191.58	88.34	279.17	57.47
4-Ethylphenol	1.181	0.280	80.61	192.29	88.19	279.73	56.91
2-Methylphenol	1.169	0.149	80.03	193.75	87.02	280.01	56.06
5-Methyl-2-[(2*R*)-6-methylhept-5-en-2-yl]phenol(curcuphenol)	1.088	0.315	80.38	187.33	90.95	277.53	58.89
4-Pentylphenol	1.040	0.194	80.61	191.48	88.28	279.01	57.64
3,5-Dimethylphenol	1.037	0.132	82.55	192.88	88.16	280.29	58.29
4-Methylphenol	1.021	0.088	80.37	192.37	88.65	280.27	56.14
4-Isopropyl-3-methylphenol	0.968	0.117	80.72	188.23	92.72	280.21	56.55
2,4-Dimethylphenol	0.862	0.057	78.43	187.74	94.59	281.58	52.88
2,5-Dimethylphenol	0.847	0.066	79.89	188.49	92.96	280.70	55.23
2,6-Dihydroxybenzoic acid	0.764	0.057	89.66	197.77	68.69	265.71	79.99
3,5-Di-*tert*-butyl-4-hydroxybenzaldehyde	0.595	0.163	75.12	189.87	75.72	264.84	66.32
2-*tert*-Butyl-4,6-dimethylphenol	0.525	0.117	76.32	180.96	101.32	281.53	50.82
1-(2-Hydroxy-4-methylphenyl)ethanone	0.504	0.049	89.34	196.98	75.09	271.31	74.07
2,6-Dimethylphenol	0.484	0.068	78.12	189.73	92.60	281.58	52.58
2-Hydroxy-3-methylbenzoic acid(3-methylsalicylic acid)	0.371	0.064	90.54	197.34	77.36	273.95	72.63
2-(6-Hydroxy-6-methylheptan-2-yl)-5-methylphenol(curcudiol)	0.263	0.014	81.18	181.82	92.51	273.58	63.64
2-*tert*-Butyl-4-methylphenol(2-*tert*-butyl-*p*-cresol)	0.249	0.128	78.22	184.91	96.07	280.23	54.03
2-Hydroxybenzoic acid(salicylic acid)	0.248	0.144	93.26	204.24	69.72	273.20	76.10
2,4-Di-*tert*-butylphenol	0.125	0.030	78.51	183.35	96.82	279.42	55.13
2-*tert*-Butylphenol	0.114	0.032	79.93	191.35	88.06	278.66	57.31
2,6-Di-*tert*-butylphenol	0.007	0.008	77.50	182.96	96.02	278.23	55.30
2,6-Di-*tert*-butyl-4-methylphenol	0.003	0.003	75.90	177.18	103.30	279.72	52.22
2,4,6-Tri-*tert*-butylphenol	0.002	0.003	76.17	175.69	103.89	278.83	53.37
Benzaldehyde	0.002	0.002	75.11	220.84	93.35	313.44	17.71
2,4-Dimethoxybenzaldehyde	0.001	0.001	76.34	194.88	114.58	308.71	23.67
1-(4-Hydroxy-3-methoxyphenyl)ethanone	5.102	0.395	79.39	190.77	78.83	268.84	66.59
4-Methylbenzene-1,2-diol(4-methylcatechol)	4.419	0.114	75.10	186.23	87.99	273.47	57.67
(*E*)-3-(4-Hydroxyphenyl)prop-2-enoic acid(coumaric acid)	3.766	0.146	81.86	198.03	61.83	259.10	78.79
4-Hydroxybenzaldehyde	3.646	0.448	75.02	207.03	57.55	263.83	67.23
(*E*)-3-(4-Hydroxy-3-methoxyphenyl)prop-2-enoic acid(*trans*-ferulic acid)	3.474	0.176	78.02	189.25	74.12	262.62	71.44
4-Hydroxybenzoic acid	3.299	0.235	85.38	209.43	52.72	261.40	80.01
(*E*)-3-(3-Hydroxyphenyl)prop-2-enoic acid(*m*-hydroxycinnamic acid)	2.690	0.005	83.11	203.35	66.51	269.11	70.03
4-Hydroxy-3,5-dimethoxybenzaldehyde(syringaldehyde)	2.591	0.413	72.37	186.88	79.36	265.49	62.91
2-Methoxyphenol	2.495	0.064	78.46	186.67	96.55	282.47	52.02
2,6-Dimethoxyphenol	2.177	0.160	74.49	181.16	98.36	278.77	51.76
1,3-Benzodioxol-5-ol(sesamol)	2.107	0.070	77.54	185.26	88.90	273.41	60.16
4-Allyl-2-methoxyphenol (eugenol)	1.811	0.118	77.70	181.77	100.17	281.19	52.55
4-Prop-2-enylphenol(4-allylphenol)	1.804	0.103	80.96	192.57	85.38	277.20	59.79
2-Methoxy-4-methylphenol	1.790	0.093	77.38	181.32	103.61	284.18	49.23
4-*tert*-Butylphenol	1.512	0.108	80.60	190.32	89.93	279.50	57.14
4-(2-Hydroxyethyl)phenol(tyrosol)	1.131	0.167	81.50	191.92	83.92	275.09	62.45
3,4-Dimethylphenol	0.852	0.101	80.44	189.15	92.60	281.00	55.47
2-Methyl-5-propan-2-ylphenol(carvacrol)	0.829	0.259	79.73	187.22	94.06	280.52	55.24
5-Methyl-2-propan-2-ylphenol(thymol)	0.545	0.152	80.20	187.91	92.41	279.57	56.67
1-(2-Hydroxyphenyl)ethanone(*o*-hydroxyacetophenone)	0.404	0.141	88.83	198.52	82.60	280.37	64.50
2,6-Di-*tert*-butyl-4-ethylphenol	0.005	0.001	76.12	177.07	102.86	279.17	52.98

Phenol (H-ORAC value = 1.76 mol TE mol^−1^) was used as the standard for discussion to confirm the variation in the H-ORAC values with the type and position of the substituents. The results obtained for benzaldehyde (0.00) and 2,4-dimethoxybenzaldehyde (0.00) indicated that it was not possible to measure the antioxidant capacities of aromatic compounds without phenolic groups using the H-ORAC method. However, it was found that the H-ORAC values of phenolic compounds bearing alkyl, bulky alkyl, carbonyl, hydroxyl, and methoxy groups were affected by the electronic and steric properties of these substituents, and their effects were exerted at the position of substitution.

More specifically, it was found that the presence of an alkyl group at the *para* position of the benzene ring decreased the H-ORAC value, with 4-methylphenol (1.02), 4-ethylphenol (1.18), 4-propylphenol (1.31), 4-butylphenol (1.23), 4-pentylphenol (1.04), tyrosol (1.13), and 4-*tert*-butylphenol (1.51) exhibiting lower H-ORAC values than phenol itself, thereby suggesting that the electron-donating and water-soluble properties of these compounds may be related to the H-ORAC value. Furthermore, the values obtained for 2-methylphenol (1.17), 3-methylphenol (1.64), 4-methylphenol (1.02), 2,4-dimethylphenol (0.86), 3,5-dimethylphenol (1.04), 2,5-dimethylphenol (0.85), and 3,4-dimethylphenol (0.85) showed that the introduction of additional methyl groups further decreased the H-ORAC value.

These results indicate that *para*-substitution resulted in only a slight steric effect from bulky alkyl groups; however, substitution at the *ortho* position led to significantly lower H-ORAC values. For example, the H-ORAC value of phenol (1.76) slightly decreased only slightly in the case of 4-*tert*-butylphenol (1.51), but was significantly lower in the case of 2-*tert*-butylphenol (0.11). This feature was also observed for 2,4-di-*tert*-butylphenol (0.12), 2-*tert*-butyl-*p*-cresol (0.25), thymol (0.54), curcuphenol (1.09), and curcudiol (0.26). This enhanced reduction was attributed to the increased steric hindrance at the phenolic OH group, which is the point of reaction for these antioxidants. In addition, the decreased H-ORAC values for carvacrol (0.83) and 4-isopropyl-3-methylphenol (0.97) also appeared to correlate with their electron-donating and water-soluble properties. Furthermore, significantly reduced H-ORAC values were observed for 2,6-dimethylphenol (0.48), 2,4,6-tri-*tert*-butylphenol (0.00), 2,6-di-*tert*-butyl-4-methylphenol (0.00), 2,6-di-*tert*-butylphenol (0.01), 2,6-di-*tert*-butyl-4-ethylphenol (0.00), and 2-*tert*-butyl-4,6-dimethylphenol (0.53), wherein both *ortho* positions were substituted, further confirming the involvement of the phenolic OH group in the reaction.

In contrast, our results indicated that the presence of a carbonyl group at the *para* position increased the H-ORAC value, owing to the electron-withdrawing nature of these substituents; however, the reverse effect was observed for carbonyl substitution at the *ortho* position. This was attributed to the fact that these *ortho*-substituted compounds formed intramolecular hydrogen bonds in their most stable structures, which reduced the reactivity of the phenolic OH group due to the energy required to break the hydrogen bond. 2-Hydroxybenzoic acid (0.25), *o*-hydroxyacetophenone (0.40), 1-(2-hydroxy-4-methylphenyl)ethanone (0.50), 2,6-dihydroxybenzoic acid (0.76), and 3-methylsalicylic acid (0.37) are typical examples of compounds substituted with a carbonyl group at the *ortho* position. However, it should be noted that 2-hydroxybenzaldehyde (2.65) is an exception to the above rule due to its stable structure. Additionally, the C–H bond dissociation energy of the aldehyde group in 2-hydroxybenzaldehyde is 79.00 kcal mol^−1^, which is smaller than that of phenol itself (82.35 kcal mol^−1^).

Compounds with a catechol or hydroquinone structure (*i.e.*, 1,2-benzenediol and 1.4-benzenediol) exhibited significantly higher H-ORAC values of 3.97 and 11.61, respectively. These compounds are characterized by their ability to capture a ROO˙ radical to form quinone structures. In addition, the H-ORAC value of *p*-benzoquinone is higher than that of *o*-benzoquinone due to the fact that the hydroxyl groups of *o*-benzoquinone are hydrogen-bonded to one another. Additionally, *p*-benzoquinone is more stable than *o*-benzoquinone. However, it should be noted that *meta* substitution (*i.e.*, for 1,3-benzenediol, 1.94) did not lead to any significant improvement in the H-ORAC value due to the absence of a stable quinone form.

It was also found that the presence of a methoxy group increased the H-ORAC value compared to that of phenol, as observed for 2-methoxyphenol (2.50), 3-methoxyphenol (3.08), and 4-methoxyphenol (2.85). This increase can be attributed to radical dissociation of the phenolic hydroxyl group, followed by cleavage of the weakly bound PhO–Me bond; the BDE for this bond was determined to be 48.02 kcal mol^−1^ based on calculations carried out at the B3LYP/6-311+g(d,p) level of theory.^27^

**Scheme 1:**
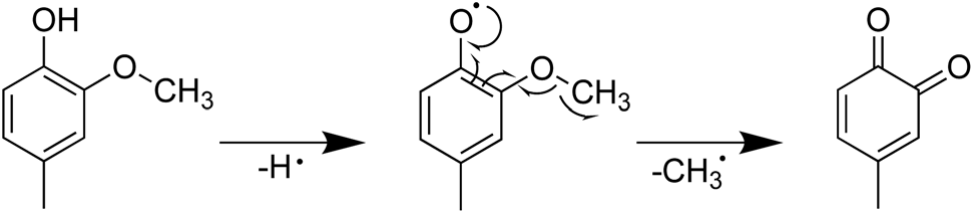
Mechanism for the reaction between 2-methoxy-4-methylphenol and ROO˙.

**Scheme 2:**
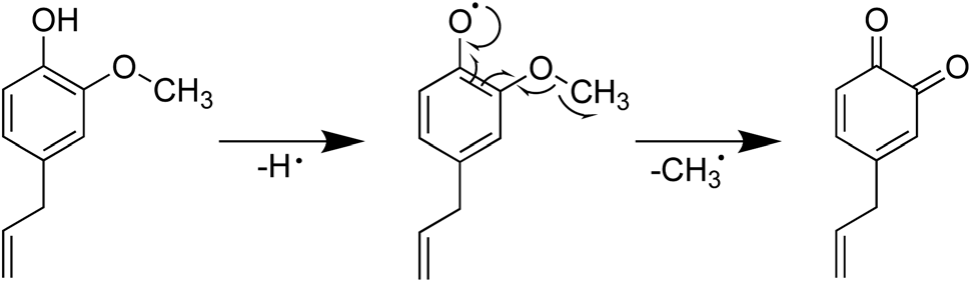
Mechanism for the reaction between eugenol and ROO˙.

**Scheme 3:**
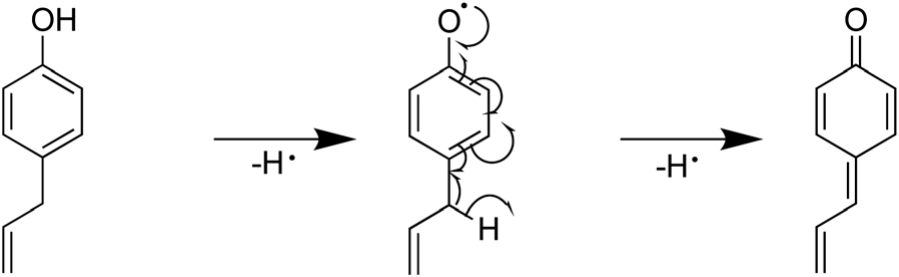
Mechanism for the reaction between 4-allylphenol and ROO˙.

Furthermore, the introduction of an allyl group on the benzene ring led to a higher H-ORAC value, as can be seen by comparing the values for 4-methylphenol (1.02) and 4-allylphenol (1.80). This comparison also showed that the influence of the allyl group dominated over the reducing effect of the alkyl group. Eugenol (or 4-allyl-2-methoxyphenol) also gave a high H-ORAC value (1.81); however, in this case, no enhanced effect was observed due to the presence of both methoxy and allyl groups, likely due to the reaction of only one of these two groups, as outlined in Schemes 1–3. As presented in Schemes 1 and 2, eugenol and 2-methoxy-4-methylphenol can capture a ROO˙ radical and subsequently cleave the methoxy group to form an *o*-quinone; the generated methyl radical can also capture the ROO˙ radical. Furthermore, since the BDE of allyl group of 4-allylphenol is small, the hydrogen radicals generated during bond cleavage can capture the additional ROO˙ (Scheme 3). However, it should be noted that the allyl group of eugenol is not cleaved due to the fact that the *o*-quinone produced after cleavage of the methoxy bond does not possess the phenolic hydroxyl group required to initiate the reaction.

## 4. Conclusions

In summary, we investigated the trend in H-ORAC values from the structures of 72 readily available derivatives of antioxidants containing phenolic hydroxyl groups. Notably, we obtained the largest amount of data in the component analysis of ORAC within this single study. In addition, we followed the method provided by Watanabe *et al.* and confirmed its reproducibility by interlaboratory collaborative studies. It should therefore be possible to compare the component analyses using the method suggested by Watanabe *et al.* in the future. Furthermore, we explored the relationship between the five indices calculated by PM7 and demonstrated that no single index could explain the results. Moreover, it should be noted that the ORAC is difficult to predict because it simultaneously measures the titer and persistence, and produces an index from the area. Furthermore, we determined the trends between different substituents and the resulting ORAC values through further analysis by combining substituents as experimental data. We therefore expect that our database will serve as a new benchmark and tool for molecular design. Although we focused on phenols for the purpose of this study, other antioxidants are currently being examined in our laboratory, and the results of these evaluations will be reported in due course.

## Author contributions

All authors contributed to the project, and the main contributions were as follows: conceptualization, SS and HG; methodology, SS; validation, SS and AK; investigation, SS and AK; resources, HG; writing – original draft preparation, SS; writing – review and editing, HG; visualization, SS; supervision, HG; project administration, HG; funding acquisition, HG All authors have read and agreed to the published version of the manuscript.

## Conflicts of interest

There are no conflicts to declare.

## Supplementary Material
